# In-Depth Characterization of Sheep (*Ovis aries*) Milk Whey Proteome and Comparison with Cow (*Bos taurus*)

**DOI:** 10.1371/journal.pone.0139774

**Published:** 2015-10-08

**Authors:** Minh Ha, Manya Sabherwal, Elizabeth Duncan, Stewart Stevens, Peter Stockwell, Michelle McConnell, Alaa El-Din Bekhit, Alan Carne

**Affiliations:** 1 Department of Biochemistry, Otago School of Medical Sciences, University of Otago, Dunedin, New Zealand; 2 Department of Oral Sciences, School of Dentistry, University of Otago, Dunedin, New Zealand; 3 Laboratory for Evolution and Development, Genetics Otago & Gravida; National Centre for Growth and Development, Department of Biochemistry, University of Otago, Dunedin, Aotearoa-New Zealand; 4 Department of Microbiology and Immunology, Otago School of Medical Sciences, University of Otago, Dunedin, New Zealand; 5 Department of Food Science, University of Otago, Dunedin, New Zealand; Nanjing Medical University, CHINA

## Abstract

An in-depth proteomic study of sheep milk whey is reported and compared to the data available in the literature for the cow whey proteome. A combinatorial peptide ligand library kit (ProteoMiner) was used to normalize protein abundance in the sheep whey proteome followed by an in-gel digest of a 1D-PAGE display and an in-solution digestion followed by OFFGEL isoelectric focusing fractionation. The peptide fractions obtained were then analyzed by LC-MS/MS. This enabled identification of 669 proteins in sheep whey that, to our knowledge, is the largest inventory of sheep whey proteins identified to date. A comprehensive list of cow whey proteins currently available in the literature (783 proteins from unique genes) was assembled and compared to the sheep whey proteome data obtained in this study (606 proteins from unique genes). This comparison revealed that while the 233 proteins shared by the two species were significantly enriched for immune and inflammatory responses in gene ontology analysis, proteins only found in sheep whey in this study were identified that take part in both cellular development and immune responses, whereas proteins only found in cow whey in this study were identified to be associated with metabolism and cellular growth.

## Introduction

Milk proteins are widely regarded as a functional food with many nutritional and health promoting benefits [1]. Worldwide, milk production from small ruminants such as sheep and goats is marginal in comparison with that of cow milk. However, in several countries, such as in the Mediterranean where the climate and environment is more suitable for small ruminants rather than cows [2], small ruminant milk production is significant. In addition, small ruminant milks appear to be potentially less allergenic and have become important as a substitute for people (including children) with cow milk intolerance [3]. Sheep milk has a relatively high protein content and is often used for specialty cheese production [4]. Although milk production in New Zealand is dominated by cows, there is considerable interest in promoting the expansion of sheep dairy. The by-product whey fraction from sheep milk cheese manufacture is currently under-utilized with land-spreading being a common method of disposal [5]. The proteome of cow milk and milk whey has attracted extensive research [6, 7], whereas knowledge of the sheep milk whey proteome is substantially limited in comparison [8]. Sheep milk whey proteins themselves are increasingly being recognized for their bioactivity or health-promoting benefits, such as immunomodulatory, antimicrobial and transfer of passive immunity activities [9]. In addition, natural digestion of sheep whey proteins in the gastrointestinal tract can generate peptides with a variety of bioactivities, for instance antihypertensive, opioid, antibacterial, antioxidant, and immunomodulatory activities [10–12]. In-depth identification of protein constituents in the milk whey proteome is therefore important for a more detailed understanding of the biological significance of milk whey, in the temporal production of milk, and to compare differences between species.

Previous studies of milk whey proteomes have focused on various methods including electrophoresis (notably 2D-PAGE), and multi-dimensional liquid chromatography, combined with mass spectrometry workflows [8, 13–15]. The depth of proteomic analysis using these methods has been limited by the wide dynamic range of protein abundance in whey. The major whey proteins, including β-lactoglobulin (not present in human), α-lactalbumin, serum albumin, lactoferrin, lactoperoxidase and immunoglobulins, constitute over 90% of the total protein present in whey [9]. The minor whey proteins are attracting increasing interest, as it is known that proteins derived from serum, and from infiltrating neutrophils and lymphocytes are secreted into milk resulting in transfer of health-promoting components, including those contributing to reducing infection rates in infants [16]. More recently, proteomic analysis of cow and human whey using a ProteoMiner Protein Enrichment combinatorial peptide ligand library kit to normalize protein abundance and hence compress the dynamic range, prior to use of conventional fractionation procedures, has enabled increased depth of analysis [17, 18].

In the current study, sheep acid whey fraction was subjected to protein abundance normalization using a ProteoMiner kit, followed by both in-gel digest 1D-PAGE/LCMS/MS and in-solution digest OFFGEL IEF/LCMS/MS. By using a combination of PAGE and OFFGEL IEF combined with LCMS/MS in this analysis, a substantially larger number of whey proteins have been identified compared to a previous study on sheep whey using 2D-PAGE [8], and on cow whey using 2D-LC [7, 14]. Gene ontology (GO) analysis of the sheep proteome generated in this study revealed that proteins associated with immune and inflammatory responses, and cellular development are significantly enriched within the sheep whey fraction. Comparison of the sheep whey in-depth proteome generated in this study with the cumulative data currently available for cow whey identified differences in biological processes and molecular functions in which whey proteins of the two species are involved, reflecting differences in physiological requirements of offspring of the two species.

## Materials and Methods

### Materials

All chemicals were obtained from Sigma Aldrich, Auckland, New Zealand (NZ), unless otherwise stated.

### Methods

#### Milk Sample Collection

No ethical approval was required for this study. The farm (Blue River Dairy, 111 Nith Street, Invercargill 9812, New Zealand) from which the milk was obtained is a regular dairy milking farm supplying milk to the local market. The owner of the farm gave permission for milk collected to be used in this study. Milk was simply collected from healthy animals with no special interventions with the milking sheep. The study did not involve endangered or protected species.

Milk samples were collected and processed according to a method previously described [19, 20]. Unpasteurized sheep whole milk was obtained from three healthy East Friesian sheep in mid-lactation (2 months postpartum). The sheep were selected on the basis of clinical examination with normal weaning performance and an absence of visible infectious diseases, no signs of bacterial infection and non-compromised pregnancy. Prior to sample collection, milk from each animal was subjected to an established method, the California Mastitis Test (somatic cell levels in milk) to screen for subclinical mastitis [21]. All milk samples were within acceptable somatic cell levels as determined by the Test. Milk from each animal was obtained by hand into pre-chilled autoclaved glass bottles and kept on ice during transportation to the lab. The milk samples were immediately pooled and centrifuged (4,000 *g*, 30 min, 4°C) to remove the cream fraction. Any pelleted (casein) protein was re-suspended and then aliquots of the 'defatted' milk were used for experiments.

#### Preparation of Acid Whey from Defatted Whole Milk

Preparation of acid whey was performed according to the method of Parris, Purcell [22]. Defatted whole milk was made to 60 mM CaCl_2_ with 1 M CaCl_2_ and then titrated to pH 4.3 with 1 M HCl. The sample was then centrifuged at 10,000 *g* for 30 min using an Allegra X-15R centrifuge (Beckman Coulter) at 4°C. The clarified supernatant (acid whey fraction) was decanted, aliquoted and stored at -20°C.

#### Sheep Whey Abundance Normalization Using a ProteoMiner Kit

Preparation of sheep acid whey for ProteoMiner treatment was performed using a previously described method with modification [19]. Sheep whey (150 mL) was made to 90% saturation with ammonium sulfate at 4°C and centrifuged at 10,000 *g* for 30 min using an Allegra X-15R centrifuge (Beckman Coulter) at 4°C to pellet precipitated whey proteins. The pellet was re-dissolved to a total volume of 25 mL with MQ-water and dialyzed in benzoylated dialysis tubing (2.0 kDa cut-off) (Sigma-Aldrich, Auckland, NZ) against 10 mM Tris-HCl, pH 7.0, 4°C, 16 h, followed by lyophilization. The lyophilized sample was re-dissolved in MQ-water to 60 mg.mL^−1^. Total protein was determined by bicinchoninic acid assay according to Smith, Krohn [23]. One mL of the above 60 mg.mL^−1^ sheep acid whey was subjected to protein abundance normalization by treatment with a ProteoMiner Large Capacity Protein Enrichment Kit (BioRad, Auckland, NZ) according to the manufacturer’s instructions. The enriched whey protein material eluted from the ProteoMiner column was desalted using a 2D Clean Up Kit (GE Healthcare, Auckland, NZ) and the whey protein pellets re-dissolved in 100 μL MQ-water and stored at -20°C.

#### 1D-PAGE and In-gel Tryptic Digestion

One quarter (25 μL) of the ProteoMiner enriched sheep acid whey protein fraction obtained after 2D Clean Up Kit desalting was subjected to 1D-PAGE on a 12 well Novex BOLT 4–12% bis-Tris electrophoresis gel run in a Novex BOLT mini-gel electrophoresis system (Life Technologies, Auckland, NZ). The protein sample was added to Novex BOLT LDS sample buffer (4X) and Novex BOLT sample reducing agent (10X) according to supplier's recommendations, prior to electrophoresis. Novex Sharp Pre-Stained Protein Standard (Life Technologies, Auckland, NZ) was run in one lane for calibration. After electrophoresis the gels were washed in MQ-water 3 x 5 min and then stained with Simply Blue SafeStain (Invitrogen, Auckland, NZ) according to supplier's instructions. The whole gel lane containing the ProteoMiner enriched sheep acid whey fraction was excised from the gel and then divided into 6 segments prior to in-gel tryptic digestion. Each gel lane segment was subjected to in-gel digestion with trypsin using a robotic workstation for automated protein digestion (DigestPro Msi, Intavis AG, Cologne, Germany). The protocol for automated in-gel digestion was based on the method of Shevchenko, Jensen [24]. Eluted peptides were dried using a Savant Speed Vac (Savant, France) centrifugal concentrator, prior to LCMS/MS.

#### OFFGEL Isoelectric Focusing

One half (50 μL) of the ProteoMiner enriched sheep acid whey protein fraction obtained after 2D Clean Up Kit desalting was diluted with 300 μL freshly prepared 100 mM ammonium bicarbonate and subjected to in-solution digestion with trypsin, 37°C, 16 h. The tryptic hydrolysate was desalted using a C18 SepPak cartridge (Waters, MA, USA) and then dried using a Savant Speed Vac (Savant, France) centrifugal concentrator and then dissolved in 3.7 mL MQ-water containing 4.8% (v/v) glycerol ready for OFFGEL IEF. OFFGEL IEF was conducted on a 3100 OFFGEL Fractionator (Agilent Technologies, CA, USA) according to manufacturer's recommendations with slight modification. A 24 cm pH 3–10L Immobiline Drystrip IEF strip (GE Healthcare, Auckland, NZ) was assembled with a 24 well hopper unit in the OFFGEL apparatus. The IEF strip was rehydrated by addition of 40 μL per well of MQ-water containing 4.8% (v/v) glycerol and 5% (v/v) pH 3–10L ampholine buffer concentrate (GE Healthcare) for 15 min. Paper wicks wetted in the same rehydration solution were applied to each end of the IEF strip. After IEF strip rehydration, aliquots (150 μL) of the above processed whey hydrolysate sample were loaded in each hopper well of the OFFGEL system. After addition of mineral oil to the end compartments of the OFFGEL system, the electrodes were attached and electrophoresis conducted until 100 kVh had been accumulated over 24 h. After electrophoresis, the solution in each well was harvested, retained as separate fractions, and then dried using a Savant Speed Vac (Savant, France) centrifugal concentrator, prior to LCMS/MS.

#### LCMS/MS

Samples (6 from in-gel digests of 1D-PAGE gel segments, and 24 from OFFGEL IEF fractions) were re-solubilized in 5% (v/v) acetonitrile, 0.2% (v/v) formic acid in water and aliquots were injected onto an Ultimate 3000 nano-flow uHPLC-System (Dionex, CA, USA) that was in-line coupled to the nanospray source of a LTQ-Orbitrap XL hybrid mass spectrometer (Thermo Scientific, San Jose, CA, USA). Peptides were separated on an in-house packed emitter-tip column (75 μm ID PicoTip fused silica tubing (New Objective, Woburn, MA) packed with C–18 material on a length of 8–9 cm), using 0.2% formic acid in MQ-water (buffer A) and 0.2% formic acid in acetonitrile (buffer B). Each of the peptide fractions was preliminary analyzed with a gradient developed from 5% (v/v) acetonitrile, 0.2% [v/v] formic acid to 99% [v/v] acetonitrile, 0.2% [v/v] formic acid in water at a flow rate of 200–500 nL.min^−1^.

A typical instrument setting for the LTQ-Orbitrap was full MS in a mass range between m/z 300–2000, performed in the Orbitrap mass analyzer with a resolution of 60,000 at m/z 400 and an AGC target of 5e5. Preview mode for FTMS master scan was enabled to generate precursor mass lists. The strongest 5 signals were selected for CID (collision induced dissociation)-MS/MS in the LTQ ion trap at a normalized collision energy of 35% using an AGC target of 2e4 and one microscan. Dynamic exclusion was enabled with 2 repeat counts during 30 sec and an exclusion period of 180 sec. Exclusion mass width was set to 0.01. After the initial LCMS/MS analysis of each of the 6 samples from 1D-PAGE and the 24 fractions from OFFGEL IEF, the LCMS/MS was repeated twice with different LC gradients (5% B hold 0–6 min, to 10% B over 6–9 min, to 27% B over 9–36 min, to 40% B over 36–41 min, to 99% B over 41–44 min) and (5% B hold 0–11 min, to 25% B over 11–65 min, to 40% B over 65–75 min, to 99% B over 75–80 min), to optimize peptide fractionation and maximize depth of peptide MS analysis by allowing for bias in hydrophobicity and complexity of the peptides in multiple fractions.

#### Data Analysis

Tandem mass spectral data were generated with Proteome Discoverer 1.3 software (Thermo Scientific) using default settings with the exception of a maximum peptide length of 8000 Da. Peak lists were searched against the SheepProt1 amino acid sequence database (NCBI amino acid sequence database subset matching the genus *Ovis* which contained 31,239 sequences and was downloaded on July 5^th^ 2013) using the three search engines Sequest HT, Mascot and MS Amanda. The search was set up for full tryptic peptides with a maximum of 2 missed cleavage sites. Dynamic modifications: carbamidomethyl C, oxidized M, deamidated N and Q were included. Search parameters specified an initial MS precursor mass tolerance of 10 ppm and an MS/MS fragment tolerance of 0.8 Da. The Percolator algorithm [25] was used for FDR calculation using a cutoff of q = 0.01. In addition the following search engine threshold scores were used: Mascot ion score ≥20, MS Amanda score ≥100 and Sequest HT charge state dependent score of 2.4 (2+), 2.8 (3+), 3.2 (4&5+), 3.6 (all others).

The full sequences of milk whey proteins were retrieved from the NCBI database from available accession numbers. In parallel, the bovine and human RefSeq data in GenBank and FastA format were downloaded from the NCBI database (*ftp*.*ncbi*.*nlm*.*nih*.*gov/refseq/*). An equivalent table of protein bovine and human accession number to gene symbol was generated. Subsequently BlastP was used to match the full sequences of the identified protein (query argument) to either the human or bovine RefSeq homologue (subject argument). The BlastP searches were conducted on the cluster at the Department of Biochemistry, University of Otago, using binaries from the NCBI website [26]. The BlastP output in tabular format (outfmt argument set to 6) was used to find the best match and to retrieve official gene symbol. Duplicates were then removed from the lists of gene symbols (*sort | uniq* Bash commands) and cross-tabulated twice in an Excel spreadsheet package (Microsoft Corporation, Redmond, WA). The list of genes was converted in a presence/absence table of each gene (*Countif* function), and summarized as a tally of the co-presence of the genes (*concatenate* function, *pivot table* tool).

Identified sheep whey proteins were analyzed with gene ontology (GO) through the Database for Annotation, Visualization and Integrated Discovery (http://david.abcc.ncifcrf.gov/) [27, 28]. GO term enrichment is considered significant for a Benjamini corrected enrichment score of less than 0.05.

## Results and Discussion

### ProteoMiner treatment of sheep whey achieved significant enrichment of minor proteins

**Fig 1:**
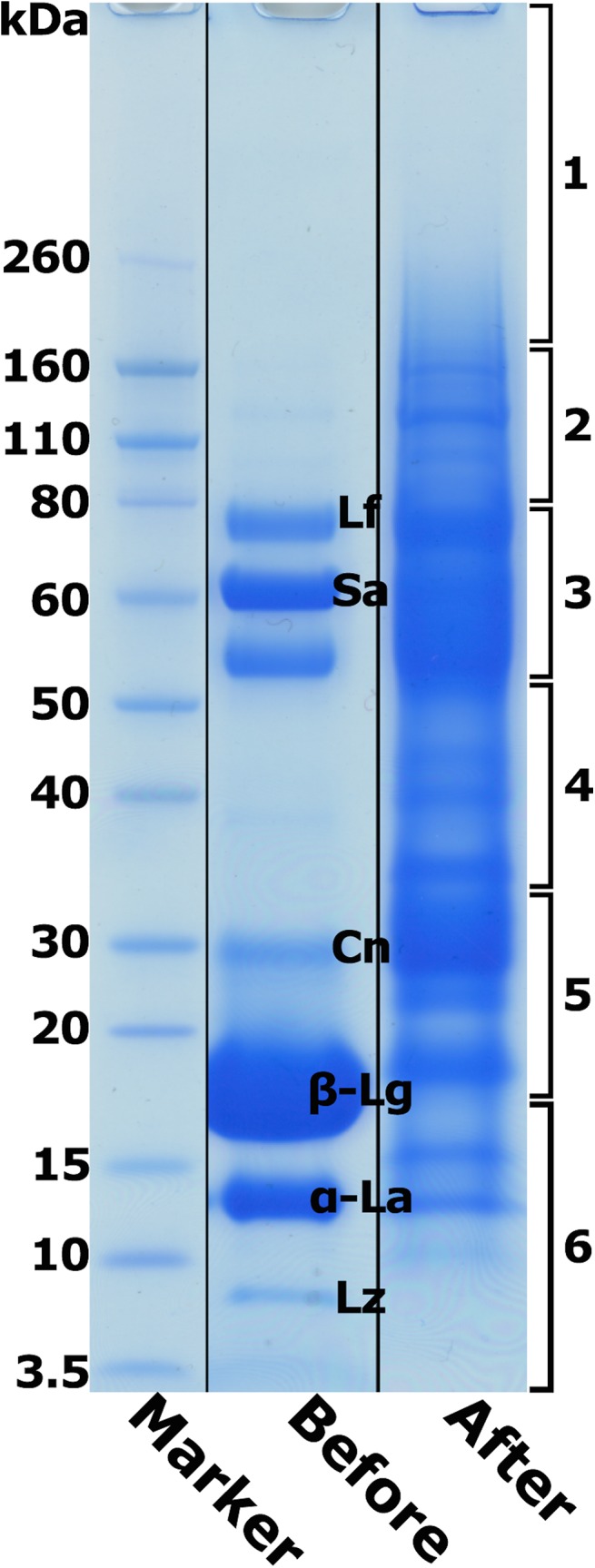
1D-PAGE of sheep acid whey before and after ProteoMiner treatment. Sheep acid whey was subjected to ammonium sulfate precipitation, desalting by 2 kDa cut-off dialysis and concentration to 60 mg.mL^−1^ prior to treatment with a ProteoMiner kit according to the manufacturer’s instructions. The ProteoMiner enriched protein fraction was desalted using a 2D Clean Up Kit and one quarter of the material was separated by 1D-PAGE using a 12 well Novex BOLT 4–12% bis-Tris electrophoresis gel. Novex Sharp Pre-Stained Protein Standards (Life Technologies, Auckland, NZ) were run in one lane for calibration. The gel was stained with Simply Blue SafeStain (Invitrogen, Auckland, NZ). After staining, the Proteominer enriched sample whole gel lane was excised, cut into six segments as indicated and subjected to an in-gel digest/ LCMS/MS workflow. Lf, lactoferrin; Sa, serum albumin; Cn, casein; β-Lg, beta-lactoglobulin; α-La, alpha-lactalbumin; and Lz, lysozyme.

Milk contains a large number of proteins with a significant dynamic range of abundance. The casein fraction (including α_s1_-, α_s2_-, β- and κ-caseins) constitutes about 80% of the total protein in sheep milk. The major whey proteins, including β-lactoglobulin, α-lactalbumin, serum albumin, lactoferrin, lactoperoxidase and immunoglobulins (indicated in Fig 1 as reported previously [29]), account for the majority of the rest of the total milk protein content [2]. Previous proteomic studies employing different method strategies with cow milk have collectively identified, apart from the major proteins, over 700 low abundance proteins in cow whey [6, 7, 14, 30]. The large dynamic range of protein abundance has hindered conventional protein identification approaches, such as ion exchange chromatography and 2D-PAGE coupled with mass spectrometry. A recent proteomic study of whey from sheep colostrum (milk produced during late pregnancy) using 1D-PAGE coupled with LCMS/MS identified 343 proteins [31]. In a previous proteomic study comparing human colostrum with late lactation human milk considerable differences in protein complement were found [18], indicating the important role played by colostrum in early infant development. More recently the use of the ProteoMiner enrichment procedure prior to conventional fractionation methods has been reported to increase the depth of protein identification in cow and human milk [17, 19]. However, it has also been reported that some proteins may have a relatively low binding affinity for the ProteoMiner combinatorial peptide ligand library and may not be represented in the enrichment [32]. In our study it is apparent that some proteins, possibly many, bind to more than one hexapeptide ligand as it appears some proteins are still relatively over-represented after performing the ProteoMiner enrichment (Fig 1). However, it is clear that the ProteoMiner enrichment generates a significant compression of the dynamic range, enabling a much greater in-depth proteomic analysis. This is consistent with previous studies in which minor proteins in defatted milk such as galectin–3 binding protein, fatty acid synthase, and actin were significantly enriched, and an even greater enrichment of these minor proteins was achieved when skim milk was depleted of caseins [17, 19].

### Two electrophoresis fractionation methods following protein abundance normalization increased depth of identification of sheep whey proteins

A 1D-PAGE separation of ProteoMiner enriched sheep whey in six segments, subjected to an in-gel digest/LCMS/MS workflow, generated 483 protein identities (S1A Table) after the LCMS/MS data were searched against the NCBInr sheep protein database. The 1D-PAGE gel lane was cut into segments in an attempt to maximise the fractionation by segregating apparent higher protein abundance regions of the gel away from those of lower abundance. It was appreciated that the limited resolution of the 1D-PAGE and also the processing of the gel lane would likely result in loss of some of the lower mass proteins, and would limit the extent of in-depth analysis. This was then followed by OFFGEL IEF separation of in-solution digested ProteoMiner enriched sheep whey into 24 fractions. After LCMS/MS of each of the 24 OFFGEL fractions the number of proteins identified was 654 (S1B Table). Combining results from 1D-PAGE and OFFEGL electrophoresis resulted in 669 identified proteins (S1C Table).

The 669 proteins in sheep whey identified here is significantly higher than that of previous individual reports of 149 proteins in cow milk whey [17], 415 in human milk [19], and 115 in human milk whey [18] in which protein abundance was normalized with a ProteoMiner kit but subsequent fractionation did not include OFFGEL fractionation. A study on sheep subclinical mastitis, employing 2D-PAGE followed by LCMS/MS of differentially expressed proteins, resulted in identification of 39 proteins in sheep milk whey and 140 in a milk fat globule membrane fraction [8, 33].

### Sheep whey proteins are involved extensively in immunological and inflammatory responses

**Fig 2:**
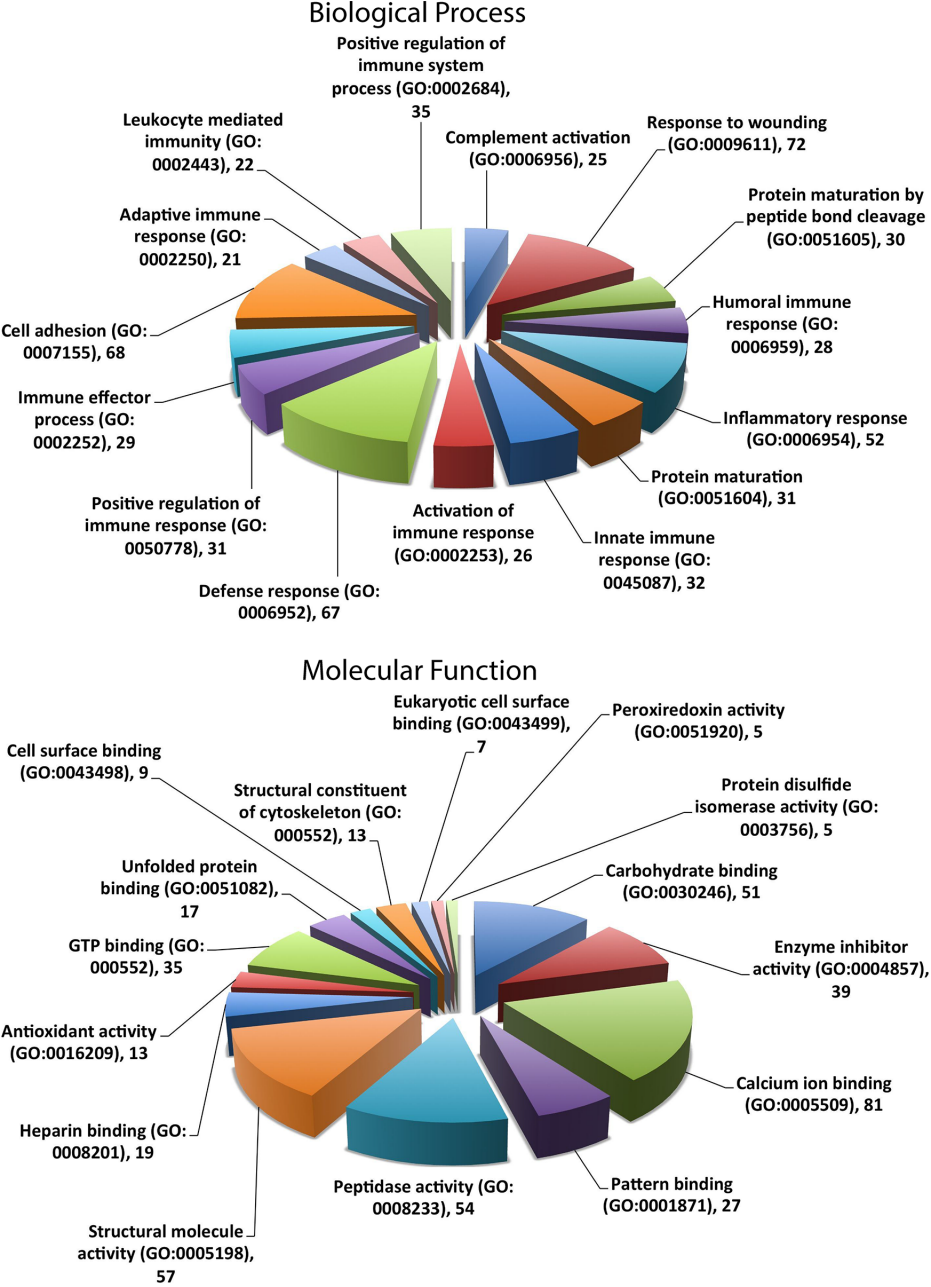
Proportions of sheep milk whey proteins involved in the top 15 GO enrichment for biological process and molecular function terms. All identified proteins in sheep milk whey were categorized with GO through the DAVID server (http://david.abcc.ncifcrf.gov/home.jsp).

Of the 669 proteins identified in sheep whey reported here, 606 were found to be from unique genes (S2 Table) and were subjected to gene ontology (GO) analysis through the Database for Annotation, Visualization and Integrated Discovery (DAVID) (http://david.abcc.ncifcrf.gov/home.jsp), using the *Homo sapiens* genome annotation as background, to obtain statistically enriched biological processes and molecular functions in which the proteins are involved (S3A and S3B Table). Fig 2 summarizes proportions of proteins involved in the top 15 enriched biological process and molecular function terms.

Proteins in sheep whey appear to be overwhelmingly involved in responses to and regulation of immunity and inflammation, for example acute inflammatory response, complement activation and innate immune response, making up 12 of the top 15 most enriched GO biological process terms with *P* values (Benjamini corrected enrichment score) from 1.68×10^−20^ to 3.63×10^−09^. These proteins are involved in a wide range of molecular functions including molecule binding, proteolysis, oxidation and antioxidant activities.

Although the protein content of milk has historically been viewed as primarily a nutritional source for infant growth and development, there has been an increasing awareness of additional health benefits. While the highly abundant casein protein group provides a principal nutritional role, the presence of antibodies, cytokines, lysozyme, lactoferrin, antimicrobial proteins and peptides, xanthine oxidase, and many other proteins in the whey fraction confer functions other than purely to nourish. Indeed, various proteins and peptides in milk have been shown to enhance solubility and absorbability of iron, zinc and calcium, benefiting bone health [34]. Furthermore, it is reported that milk provides subtle benefits throughout life, including reducing the risk of gastrointestinal infection [16] and lipid and lipoprotein metabolism complications [35].

It is also well established that milk proteins are precursors of bioactive peptides generated during digestion in the gastrointestinal tract [36]. This has resulted in growing momentum in the field of predictive bioactive peptide discovery [37, 38], which can be enhanced by in-depth proteome analyses.

Another approach in studying putative protein functions in milk is to analyze protein-protein interactions in the complex protein mixture [39]. D’Alessandro and colleagues, by compiling an exhaustive list of 573 proteins in bovine milk and 285 proteins in human milk from previous proteomic and functional studies, were able to illustrate the overall inter-relationship, as well as potential individual interactive pathways between milk proteins [6, 40]. Extensive proteomic data of sheep milk whey provides a means for further in-depth protein-protein interaction analysis of sheep milk in comparison to that of other ruminants including cow.

Due to an incomplete functional annotation of sheep proteins in the literature, functional annotation of homologous proteins in humans was used in this study with the aim of obtaining an overview of the biology of the whey proteome. However, it is appreciated that GO analysis excludes consideration of the biological significance of protein isoforms, protein complexes, or differences in post-translational modifications which have been observed in milk proteins of various species [2]. Furthermore, the use of homologous proteins from other species may lead to inaccurate reflection of the function of some proteins. Similar challenges have been acknowledged for research on other non-model organisms [39, 41].

It has been pointed out that although substantial effort has been spent on characterizing the genes in the human genome, functional annotation of human milk proteins or their homologous counterparts in other species are likely representative of the biological activities they perform in other cells or locations [42]. For example, xanthine dehydrogenase/oxidase is known to convert hypoxanthine to xanthine in most cell types. However, investigation of expression of xanthine dehydrogenase/oxidase in the mammary gland suggested that the function of this enzyme in milk is likely to reverse the endocytotic process of milk fat globule secretion [43]. The challenge in understanding gene sharing between tissues is likely applicable to sheep. Hence, further functional investigation of sheep proteins is required to obtain a better understanding of the biology of the sheep milk whey proteome. Nonetheless, GO at the current time is still considered to be the best tool and the most used to analyze proteome inter-relationships, such as between a substantial collection of proteins in milk and milk whey, to obtain an overview of the biology system and further understanding of potential benefits for human consumption [39].

### Comparison of the biology of the sheep and cow whey proteomes

Bovine milk is a major human food and agro-economical product that has attracted extensive research in the field of functional food and human nutrition. Cow milk protein-derived peptides and their precursors have been shown to provide numerous health-promoting benefits, such as regulation of the immune, cardiovascular, nervous, and gastrointestinal systems [36]. However, several cow milk proteins, e.g. β-lactoglobulin and α-casein, are considered to be more allergenic than the corresponding proteins in other species such as small ruminants. Milk protein composition is reported to vary with species, arising from differences in physiology and the varying nutritional requirements of offspring. Major proteins in sheep milk have been shown to possess differences in amino acid composition in comparison to those of cow milk [44]. Thus, in-depth comparison of the whey proteome of other species to that of cow has the potential to provide valuable information on the nutritive and health-promoting characteristics of milk proteins for development of functional foods.

**Fig 3:**
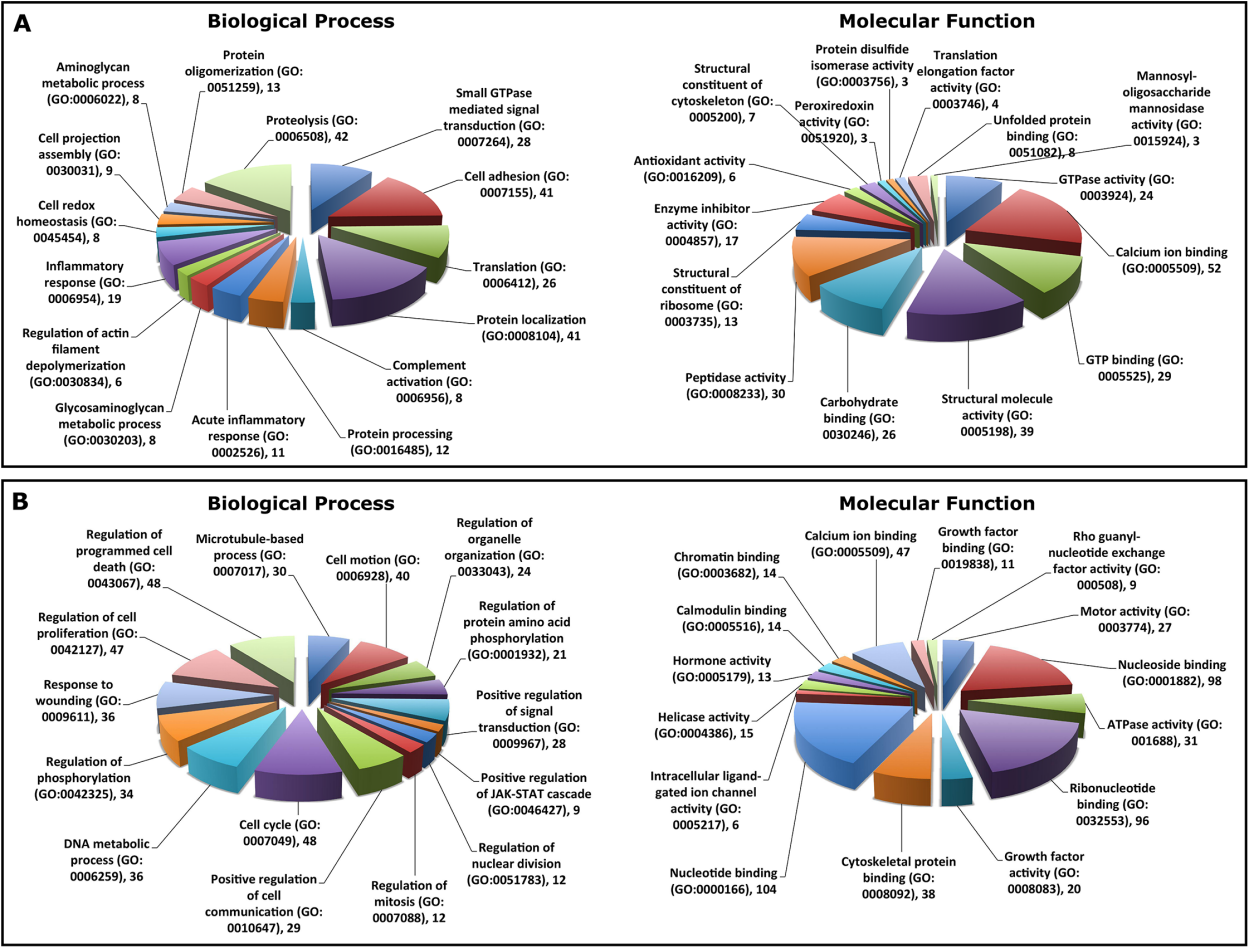
Proportion of top 15 GO term enrichment for biological process and molecular function of proteins unique to (A) sheep milk whey (373 proteins) and (B) cow milk whey (550 proteins) from the current data set. Proteins were categorized with GO through the DAVID server (http://david.abcc.ncifcrf.gov/home.jsp).

In order to compare sheep and cow whey proteomes at an in-depth level, 606 sheep whey proteins identified in this study were compared to those of a list of 783 cow whey proteins from unique genes (S2 Table) that we compiled from the literature in this study [6, 7, 13, 14, 30]. This provided an opportunity to compare a significant number of proteins from each species that likely represented similar depth of protein identity. While the two whey proteomes share 233 proteins in common, a greater number (373 for sheep and 550 for cow) appeared to be unique to each species in the current data sets. GO analysis of proteins identified exclusively in sheep and cow proteomes respectively (Fig 3) revealed that while proteins unique to cow are significantly involved in cellular growth and metabolism, those in sheep are distributed in cellular establishment, signaling, protein maturation, and inflammatory and other immune responses. GO analysis of proteins commonly identified in both species (S1 Fig) showed these proteins are mostly associated with immunity.

The high number of unmatched proteins between the two data sets was unexpected. Consequently, possible sources of error were tested, including using a different reference set of gene names for the comparisons. Specifically, in order to coherently compare the list of peptides between different species and to utilize the superior annotations present for human genes, the gene symbols encoding the human homologues (RefSeq database) were identified for both sets of peptide hits. In light of possible copy-number changes and possible gene losses in the human genome compared to the genomes of the animals analyzed here, the analysis was repeated with the difference that the matching was done to the bovine RefSeq peptide database as opposed to human. Nevertheless, the resulting comparison did not differ from that performed with the human gene symbols. Consequently, no errors arising from this approach were identified, therefore, even though surprising, the high degree of mismatch appears not to be an artifact, but a biological characteristic.

**Fig 4:**
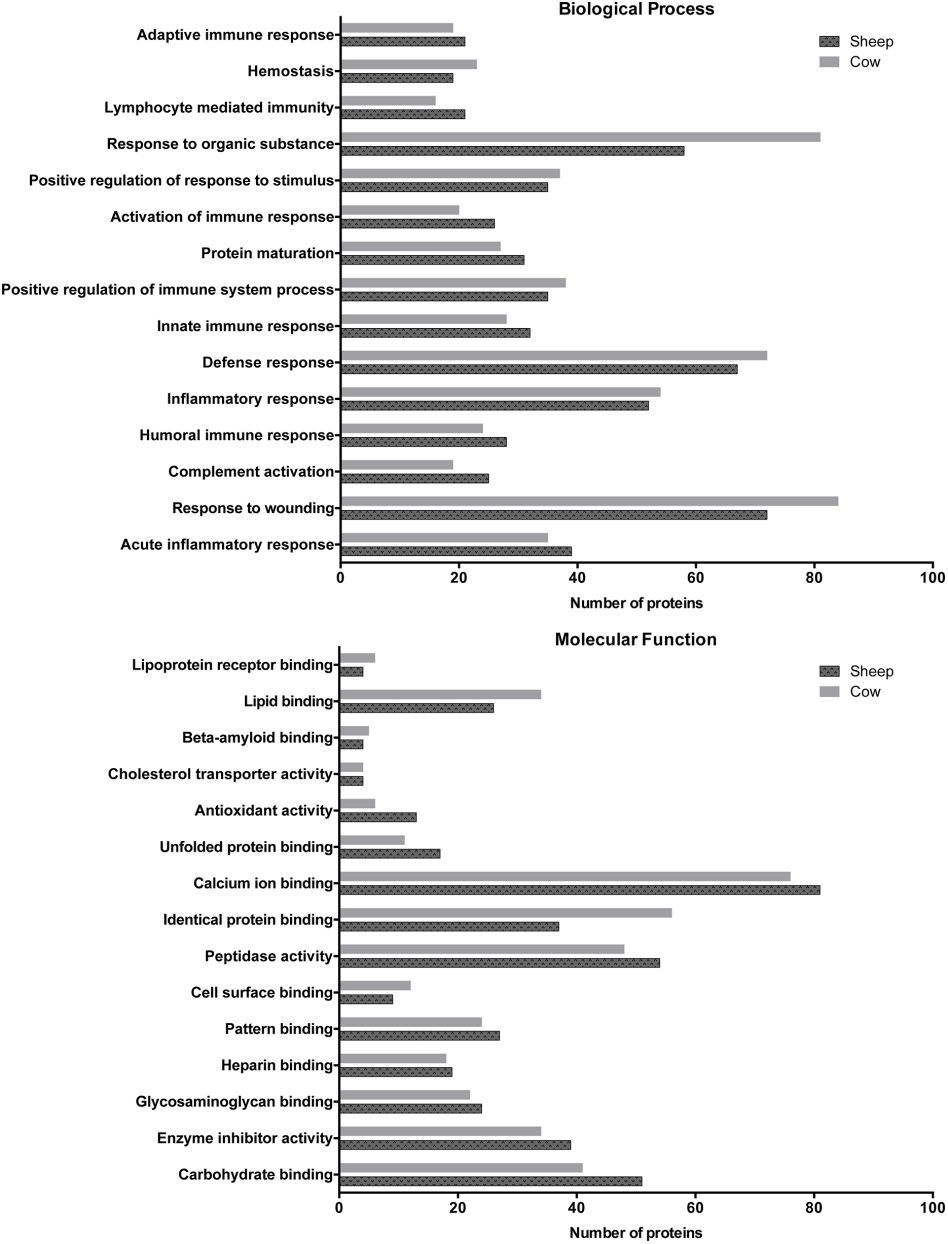
Number of milk whey gene products involved in the top 15 enriched biological process and molecular function GO terms that are shared by cow and sheep the current data sets.

Investigation of the distribution of proteins in the top 15 enriched biological process and molecular function GO terms shared by the two species (Fig 4) shows that a comparable number of sheep milk proteins are involved in most of the top 15 enriched biological process and molecular function GO terms. While sheep and cow milk whey exhibit many similar biological process and molecular function characteristics in which the same proteins can be found (S1 Fig), uniquely identified proteins associated with each of the top 15 common biological process and molecular function GO terms in cow and sheep milk whey respectively are listed in Table 1.

**Table 1:** Proteins identified only in cow milk or only sheep milk in the top 15 enriched biological process and molecular function GO terms shared by the two species.

**Biological Process**			
GO Term	Unique to Sheep	Unique to Cow	Common
Acute inflammatory response (GO:0002526)	C1QA, C1QB, C1QC, C4B, C8A, CRP, KLKB1, KRT1, MASP2, TLR4, YWHAZ	APCS, CFI, IGF2, IL1B, IL6, INS, MBL2, ORM1	A2M, AHSG, B4GALT1, C1R, C1S, C2, C3, C4A, C4BPA, C5, C6, C7, C8B, C9, CFB, CFD, CFH, CLU, F2, FN1, ITIH4, LBP, MASP1, SAA1, SERPINA1, SERPINF2, SERPING1, TF
Response to wounding (GO:0009611)	ANXA1, AZU1, C1QA, C1QB, C1QC, C4B, C8A, CD9, CRP, ITGAL, KLKB1, KRT1, LAMB2, LMAN1, MASP2, NDST1, PRDX5, PROS1, PTX3, RAC1, TIMP3, TLR4, TNFAIP6, YWHAZ	ANXA5, AOX1, APCS, APOH, CD44, CFI, CXCL2, F11, F13B, FGF2, GNAQ, IGF1, IGF2, IGFBP1, IGFBP4, IL10, IL1B, IL6, INS, MBL2, MECOM, MYH10, MYH2, NFKB1, ORM1, PDGFA, PDGFB, PLAT, PPARD, PXK, S100A12, S100A8, S100A9, SERPIND1, TGFB1, TLR7, TNF	A2M, AHSG, AOC3, B4GALT1, C1R, C1S, C2, C3, C4A, C4BPA, C5, C6, C7, C8B, C9, CD14, CD36, CFB, CFD, CFH, CLU, CTSB, DSP, F2, F9, FGA, FGB, FGG, FN1, GSN, ITIH4, KNG1, LBP, MASP1, MIA3, PEBP1, PLG, PROC, SAA1, SERPINA1, SERPINA10, SERPINC1, SERPINF2, SERPING1, SPP1, TF, TGFB2, THBS1
Complement activation (GO:0006956)	C1QA, C1QB, C1QC, C4B, C8A, CRP, KRT1, MASP2	CFI, MBL2	C1R, C1S, C2, C3, C4A, C4BPA, C5, C6, C7, C8B, C9, CFB, CFD, CFH, CLU, MASP1, SERPING1
Humoral immune response (GO:0006959)	C1QA, C1QB, C1QC, C4B, C8A, CRP, KRT1, MASP2	CFI, IL6, MBL2, TNF	C1R, C1S, C2, C3, C4A, C4BPA, C5, C6, C7, C8B, C9, CFB, CFD, CFH, CLU, DMBT1, GPI, LTF, MASP1, SERPING1
Inflammatory response (GO:0006954)	ANXA1, AZU1, C1QA, C1QB, C1QC, C4B, C8A, CRP, ITGAL, KLKB1, KRT1, MASP2, NDST1, PRDX5, PTX3, RAC1, TLR4, TNFAIP6, YWHAZ	AOX1, APCS, CD44, CFI, CXCL2, IGF2, IGFBP4, IL10, IL1B, IL6, INS, MBL2, MECOM, NFKB1, ORM1, PXK, S100A12, S100A8, S100A9, TGFB1, TLR7, TNF	A2M, AHSG, AOC3, B4GALT1, C1R, C1S, C2, C3, C4A, C4BPA, C5, C6, C7, C8B, C9, CD14, CFB, CFD, CFH, CLU, F2, FN1, ITIH4, KNG1, LBP, MASP1, SAA1, SERPINA1, SERPINF2, SERPING1, SPP1, TF, THBS1
Defense response (GO:0006952)	ANXA1, AZU1, C1QA, C1QB, C1QC, C4B, C8A, CRP, CTSG, HLA-B, ITGAL, KLKB1, KRT1, LGALS3BP, MASP2, NDST1, PGLYRP2, PRDX1, PRDX5, PTX3, RAC1, TLR4, TNFAIP6, YWHAZ	AOX1, APCS, CD44, CD5L, CFI, COTL1, CXCL2, DEFB4A, IFIH1, IFNG, IGF2, IGFBP4, IL10, IL12A, IL1B, IL4, IL6, INS, MBL2, MECOM, NFKB1, NOS2, ORM1, PXK, S100A12, S100A8, S100A9, TGFB1, TLR7, TNF	A2M, AHSG, AOC3, APOA4, B4GALT1, C1R, C1S, C2, C3, C4A, C4BPA, C5, C6, C7, C8B, C9, CAMP, CD14, CFB, CFD, CFH, CLU, CST3, DMBT1, F2, FN1, HP, ITIH4, KNG1, LALBA, LBP, LTF, MASP1, MPO, PGLYRP1, SAA1, SERPINA1, SERPINF2, SERPING1, SFTPD, SPP1, TF, THBS1
Innate immune response (GO:0045087)	C1QA, C1QB, C1QC, C4B, C8A, KRT1, MASP2, PGLYRP2, PRDX1, TLR4	CFI, IFIH1, MBL2, NOS2, TGFB1, TLR7	APOA4, C1R, C1S, C2, C3, C4A, C4BPA, C5, C6, C7, C8B, C9, CFB, CFD, CFH, CLU, DMBT1, LBP, MASP1, PGLYRP1, SERPING1, SFTPD
Positive regulation of immune system process (GO:0050778)	C1QA, C1QB, C1QC, C4B, C8A, CRP, KRT1, MASP2, TLR4, TNFSF13	CD5, CFI, IFNG, IL12A, IL1B, IL2, IL4, IL6, MBL2, NOS2, PNP, TGFB1, TLR7	ADAM10, B2M, C1R, C1S, C2, C3, C4A, C4BPA, C5, C6, C7, C8B, C9, CFB, CFD, CFH, CLU, HPX, LBP, MASP1, MIA3, PVRL2, SERPING1, TGFB2, THBS1
Protein maturation (GO:0051604)	C1QA, C1QB, C1QC, C4B, C8A, CPE, CRP, FURIN, KLKB1, KRT1, MASP2, SPCS3	APOH, CASP1, CFI, F11, LONP2, MBL2, NCLN, PTHLH	ATP6AP2, C1R, C1S, C2, C3, C4A, C4BPA, C5, C6, C7, C8B, C9, CALR, CFB, CFD, CFH, CLU, MASP1, SERPING1
Activation of immune response (GO:0002253)	C1QA, C1QB, C1QC, C4B, C8A, CRP, KRT1, MASP2, TLR4	CFI, MBL2, TLR7	C1R, C1S, C2, C3, C4A, C4BPA, C5, C6, C7, C8B, C9, CFB, CFD, CFH, CLU, MASP1, SERPING1
Positive regulation of response to stimulus (GO:0048584)	AGT, AZU1, C1QA, C1QB, C1QC, C4B, C8A, CRP, KRT1, MASP2, TLR4	CFI, EEF1E1, GHRH, IGF2, IL12A, IL1B, IL6, INS, MBL2, OSMR, PDGFB, TGFB1, TLR7, TNF	ADAM10, B2M, C1R, C1S, C2, C3, C4A, C4BPA, C5, C6, C7, C8B, C9, CFB, CFD, CFH, CLU, HPX, LBP, MASP1, PVRL2, SERPING1, TGFB2, THBS1
Response to organic substance (GO:0010033)	ACSL1, ANGPT1, ARSA, ASAH1, C1QB, CDH1, CFL1, ERO1L, HPRT1, HSPA1L, HSPA2, HSPA4, KRAS, KRT19, LEPR, LIFR, SMPD1, TGFBR3, TIMP3, TLR4	ADAM9, ADCY10, AFP, ALDOC, ANXA5, APCS, BCHE, BRCA2, CASP1, CD44, CFTR, CTSC, CTTNBP2, DGKD, DICER1, ENPP1, EPS8, FAS, GH1, GNRH1, IFNG, IGF2, IGFBP1, IGFBP2, IL10, IL12A, IL1B, IL6, INS, KDM3A, LEP, LONP2, OSMR, OXT, PDGFA, PDGFB, PRKDC, RYR1, SETD2, SLC34A2, SREBF2, TGFB1, TNF, TSHB	A2M, ACTC1, ADAM10, ADIPOQ, AHSG, ALPL, APOB, APOE, B2M, C1S, CD14, CFB, CYR61, DDR1, DNAJC3, FABP3, FBP1, GSN, HMGB2, HSP90AA1, HSP90AB1, HSPA1B, HSPA8, IDH1, LBP, LCAT, MFGE8, NME1-NME2, PEBP1, PLIN2, SDF4, SERPINA1, SERPINF2, SORT1, SPP1, TF, TGFB2, THBS1
Lymphocyte mediated immunity (GO:0002449)	C1QA, C1QB, C1QC, C4B, C8A, CRP, MASP2, PRDX1	CFI, FAS, MBL2	C1R, C1S, C2, C3, C4A, C4BPA, C5, C6, C7, C8B, C9, CLU, SERPING1
Hemostasis (GO:0007599)	CD9, KLKB1, LMAN1, PROS1	ANXA5, APOH, F11, F13B, GNAQ, IL6, PLAT, SERPIND1	C9, CD36, F2, F9, FGA, FGB, FGG, GPI, KNG1, PLG, PROC, SAA1, SERPINA1, SERPINC1, SERPING1
Adaptive immune response (GO:0002250)	C1QA, C1QB, C1QC, C4B, C8A, CRP, MASP2, TLR4	CFI, FAS, IL10, IL18BP, IL4, MBL2	C1R, C1S, C2, C3, C4A, C4BPA, C5, C6, C7, C8B, C9, CLU, SERPING1
**Molecular Function**			
**GO Term**	**Unique to Sheep**	**Unique to Cow**	**Common**
Carbohydrate binding (GO:0030246)	AZU1, CANX, CLEC2D, ENPP2, GALNT1, GALNT3, GALNT5, ITGAM, KRT1, LGALS3, LMAN1, LMAN2, MAMDC2, MAN2A1, MANBA, MLEC, NOMO1, PGLYRP2, PRG3, PTX3, SERPINA5, SOD3, TGFBR3, THBS4, TINAGL1, TNFAIP6	APCS, APOH, BGN, CD44, ENPP1, ENPP3, F11, FGF1, FGF2, IL2, MBL2, MRC1, PYGL, SERPIND1, TALDO1, ZG16B	ALDOA, APOB, APOE, ATP6AP1, CALR, CD14, CLEC3B, CYR61, FBP1, FGFBP1, FN1, FUCA1, GALM, HRG, IGF2R, KNG1, LAMC2, LPL, MAN2A2, MAN2B1, PGLYRP1, SERPINC1, SFTPD, THBS1, VTN
Enzyme inhibitor activity (GO:0004857)	AGT, ANGPTL4, ANXA1, ANXA2, C4B, COL6A3, FURIN, GCHFR, HSPA5, ITIH3, PRDX5, PROS1, SERPINA5, SERPINB3, SERPINB4, TIMP3, YWHAG	A2ML1, ANXA5, APOC3, CABIN1, CD109, CSTB, FETUB, PDE6H, SERPIND1, SPINT1, TIMP2, TRIB3	A2M, AHSG, AMBP, C3, C4A, C5, CSN2, CST3, DNAJC3, HRG, ITIH1, ITIH2, ITIH4, KNG1, PEBP1, SERPINA1, SERPINA10, SERPINB1, SERPINC1, SERPINF1, SERPINF2, SERPING1
Glycosaminoglycan binding (GO:0005539)	AZU1, ITGAM, MAMDC2, PGLYRP2, SERPINA5, SOD3, TGFBR3, THBS4, TNFAIP6	APOH, BGN, CD44, F11, FGF1, FGF2, SERPIND1	APOB, APOE, ATP6AP1, CD14, CYR61, FGFBP1, FN1, HRG, KNG1, LAMC2, LPL, PGLYRP1, SERPINC1, THBS1, VTN
Heparin binding (GO:0008201)	AZU1, ITGAM, SERPINA5, SOD3, TGFBR3, THBS4	APOH, F11, FGF1, FGF2, SERPIND1	APOB, APOE, ATP6AP1, CYR61, FGFBP1, FN1, HRG, KNG1, LAMC2, LPL, SERPINC1, THBS1, VTN
Pattern binding (GO:0001871)	AZU1, ENPP2, ITGAM, MAMDC2, PGLYRP2, PTX3, SERPINA5, SOD3, TGFBR3, THBS4, TINAGL1, TNFAIP6	APOH, BGN, CD44, ENPP1, ENPP3, F11, FGF1, FGF2, SERPIND1	APOB, APOE, ATP6AP1, CD14, CYR61, FGFBP1, FN1, HRG, KNG1, LAMC2, LPL, PGLYRP1, SERPINC1, THBS1, VTN
Cell surface binding (GO:0043498)	ATP5B, CRP	ANXA5, APOH, MBL2, PDGFA, PDGFB	ADIPOQ, APOA4, FGA, FGB, FGG, LBP, THBS1
Peptidase activity (GO:0008233)	ACE, ACY1, ANPEP, AZU1, CNDP2, CPD, CPE, CPXM1, CPXM2, CTSG, DPP3, ERAP2, FURIN, HPN, KEL, KLKB1, LNPEP, MASP2, MMP2, PDIA3, PEPD, PM20D1, PRCP, PSMA5, PSMA8, SCPEP1, SPCS3, ST14, TINAGL1, TLL1	ADAM9, ADAMTS7, AGBL3, AQPEP, CASP1, CFI, CTSA, CTSC, CTSF, CTSZ, F11, HTRA1, LGMN, LONP1, LONP2, MMP8, MMP9, PLAT, PRSS1, SPPL2A, USP24, USP28, USP34, ZFYVE9	ADAM10, C1R, C1S, C2, CFB, CFD, CPB2, CTSB, CTSD, ENO1, ERAP1, F2, F9, HP, LAP3, LTF, MASP1, PLG, PROC, PRSS22, PRTN3, PSMB1, SERPINA1, TPP1
Identical protein binding (GO:0042802)	C1QB, GLA, GPD1L, HPRT1, LMAN1, MYH9, PAK2, PCMT1, PLOD1, PON1, SHBG, TPD52L1	ACTN4, CASP8AP2, CCDC88A, CENPF, CLIP1, CTSC, DGKD, DVL1, E2F8, ENPP1, FAS, FLT1, GCC2, MYCBP2, MYO18A, NFKB1, NOS2, PDGFA, PDGFB, PYGL, ROCK1, S100B, SHMT1, TARS, TGFB1, TNF, TOP2A, TP63, TTN, WRN, ZNHIT6	ABCG2, ACTG1, ADAM10, ADIPOQ, ALDOA, AMBP, AOC3, APOA1, APOA4, APOE, ARHGDIA, B4GALT1, FBP1, FLNA, HSP90AA1, LCP1, LDHB, MASP1, NME1-NME2, PDXK, PVRL2, SDF4, SDS, TGFB2, THBS1
Calcium ion binding (GO:0005509)	ANXA1, ANXA2, ARSA, ATP5B, CACHD1, CALM1, CALU, CANT1, CANX, CDH1, CLSTN1, CRP, DCHS1, DSG1, DSG2, EDEM2, EHD4, FAM20C, FAT2, FBLN1, FURIN, GALNT1, GALNT3, GALNT5, GAS6, HSPA5, ITGAL, ITGAM, LMAN2, LTBP1, MAN1A1, MASP2, MMP2, NID1, PCDH1, PCDHB14, PCDHB7, PCDHB8, PCDHGA10, PCDHGA11, PCDHGA2, PCDHGA3, PCDHGA8, PCDHGB3, PCDHGB5, PCDHGB7, PCDHGC3, PROS1, PXDN, SULF2, THBS4, TLL1	ACTN4, AMY2B, ANO1, ANXA5, APCS, CELSR2, CRTAC1, DSC2, DST, EFHD2, EGF, FAT3, FAT4, HPSE, IDS, ITGA8, ITPR1, ITPR3, ITSN1, LRP1, MACF1, MAN1B1, MBL2, MMP8, MMP9, MRC1, NELL2, NOS2, PKDREJ, PLCB1, PLCB3, PRSS1, PTHLH, RET, RPTN, RYR1, RYR3, S100A12, S100A8, S100A9, S100B, SCUBE3, SLIT1, SPTA1, TRPC5, TTN, UTRN	AOC3, C1R, C1S, CALR, CIB1, CSN2, DAG1, F2, F9, FGG, GSN, HSP90B1, ITIH1, LALBA, LCP1, LPO, LRP2, MASP1, MPO, NUCB1, NUCB2, PDIA4, PLG, PRKCSH, PROC, SDF4, SFTPD, THBS1, TKT
Unfolded protein binding (GO:0051082)	CANX, CCT8, DNAJB11, HSPA2, HSPA5, HSPA9, LMAN1, PPIC	APCS, DNAJB9	CALR, HSP90AA1, HSP90AB1, HSP90B1, HSPA1B, HSPA8, PPIA, PPIB, SIL1
Antioxidant activity (GO:0016209)	GPX3, PRDX1, PRDX4, PRDX5, PXDN, SEP15, SOD3	N/A	ALB, APOA4, APOE, LPO, MPO, PRDX6
Cholesterol transporter activity (GO:0017127)	N/A	N/A	APOA1, APOA4, APOB, APOE
Beta-amyloid binding (GO:0001540)	N/A	BCHE	APOA1, APOE, CST3, TGFB2
Lipid binding (GO:0008289)	ALDH1A1, ANXA1, ANXA2, APOF, PITPNA, PLTP, PON1, RBP1, SHBG	ACBD3, ANXA5, APOC3, APOH, CCDC88A, CIT, DGKD, IL2, ITPR1, ITPR3, NPC2, PPARD, PREX1, PRKD3, PXK, PYGL, ROCK1	ALB, APOA1, APOA4, APOB, APOE, AZGP1, CD36, FABP3, GC, LBP, LPL, MFGE8, PEBP1, PITPNB, PSAP, PTGDS, SCGB2A2
Lipoprotein receptor binding (GO:0070325)	N/A	APOC3, LRP1	APOA1, APOB, APOE, HSP90B1

A comparison of the major proteins in milk of five different ruminants revealed that while β-lactoglobulin is the major whey protein in some species, the absence of this allergenic protein is observed in others such as human and camel [45]. In addition, the authors detected the presence of different isoforms of kappa-casein, which has been shown to be a potential allergen in different species [46]. Thus, in-depth comparison of milk between species may provide insights to sources of allergenic components.

The current data set of milk whey proteins from the two ruminant species suggests that while a comparable number of milk proteins in sheep and cow are involved with inflammatory, immune and defense responses, the composition of the proteins involved in these biological processes differ between the two species (Table 1). Of the top 15 biological process GO terms, 51 and 82 milk proteins unique to sheep and cow were identified, respectively. The identity and associated GO terms of proteins unique to sheep and cow whey are summarized in S4 and S5 Tables. The identity and associated GO terms of proteins common in whey of the two species are summarized in S6 Table. Of these proteins, those in sheep milk whey such as proteins of the complement system C1QA, C1QB, C1QC, and C8A, together with CRP, KLKB1, KRT1, MASP2, TLR4, and YWHAZ are associated with many of the top 15 biological process GO terms including acute inflammation and defense response. These proteins appear to be absent in cow milk whey. Findings in this study are consistent with previous studies in the literature in which cow milk was suggested to be almost devoid of complement in the absence of inflammation [47]. The presence of proteins of the complement system in sheep is therefore of interest for further investigation with regard to advantages of sheep milk in terms of reduced allergenicity.

Although sheep milk protein fractions, and particularly the milk fat globule membrane (MFGM) fraction, have been investigated for protein markers of presentations such as mastitis [33] as far as we are aware a definitive list of proteins associated with such presentations has not been reported, it is therefore not possible at this stage to determine whether or not the sheep milk used in this proteomics study displays markers of presentations such as mastitis. The milk samples used in this study were obtained from three individual sheep, as mentioned earlier in the Experimental Procedures section, from a local small farm where all of the sheep have been closely monitored through their life and hence to the best of our knowledge we consider that the sheep were healthy.

Evolution of milk is increasingly a subject of investigation as part of an effort to understand the function of milk constituents and their potential health benefits [48, 49]. Milk protein composition diversity illustrates that milk is species specific. Lemay and colleagues, using 197 cow milk protein genes and the mammary genomes of various species, concluded that apart from differences in protein composition that are partly due to copy number and sequence variation, most divergent proteins in milk are associated with nutritional and immunological processes [50]. Furthermore, a study of the whey proteomes of five different ruminant species reported a differential expression pattern of 211 proteins [20]. Results from the data set of the milk whey proteomes of cow and sheep in the present study confirm that, while a common core protein set is present in the two species to ensure optimal immunity for new-born, an exclusive list of proteins appears to be present in each of the two species, that likely reflects differential development of offspring physiological requirements of the two species during the course of evolution.

## Conclusions

This study contains, to our knowledge, the largest inventory of sheep whey proteins analyzed to date. Comparison of this sheep whey protein inventory with an inventory of cow whey proteins obtained from the literature, using proteomic and bioinformatic approaches, enabled a more in-depth analysis of similarities and differences in the composition of the sheep and cow whey proteomes. The significant increase in the number of proteins and protein complexes identified in sheep whey enabled a more extensive analysis of the biological significance of these proteins. Gene ontology analysis of the sheep and cow whey proteomes not only indicated that a comparable number of proteins in milk whey of the two species is involved in inflammatory, immune and defense responses, also enabled identification of unique proteins in sheep milk whey associated with various biological processes, substantiating a call for further investigation in benefits of utilization of sheep milk whey. This study therefore provides extended insight into the sheep whey proteome, in comparison to cow, with the potential to facilitate research in the design and manufacture of functional foods using sheep whey proteins, particularly in relation to utilization of whey obtained as a co-product of cheese manufacture.

## Supporting Information

S1 Fig Proportions of proteins identified in both species involved in the top 15 GO enrichment for biological process and molecular function terms. (TIF)

S1 Table S1a. 483 sheep milk whey protein entries identified by 1D-PAGE/LCMS/MS. S1b. 654 sheep milk whey protein entries identified by OFFGEL isoelectric focusing/LCMS/MS. S1c. 669 sheep milk whey protein entries identified by combining 1D-PAGE and OFFGEL isoelectric focusing methods. (XLSX)

S2 Table Proteins identified in sheep (from the current study) and cow milk whey (compiled from literature). (XLSX)

S3 Table S3a. Biological Process GO terms of sheep milk whey proteins. S3b. Molecular function GO terms of sheep milk whey proteins. (XLSX)

S4 Table Proteins unique to sheep milk whey associated with the top 15 biological process GO terms that are shared by both species. (XLSX)

S5 Table Proteins unique to cow milk whey associated with the top 15 biological process GO terms that are shared by both species. (XLSX)

S6 Table Proteins common in milk whey of the two species associated with the top 15 biological process GO terms that are shared by both species. (XLSX)
