# Supplementary figures and images for: In-Depth Characterization of Sheep (Ovis aries) Milk Whey Proteome and Comparison with Cow (Bos taurus)
Source: PLoS One. 2015 Oct 8;10(10):e0139774. doi: 10.1371/journal.pone.0139774 (PMC4598025; doi:10.1371/journal.pone.0139774)

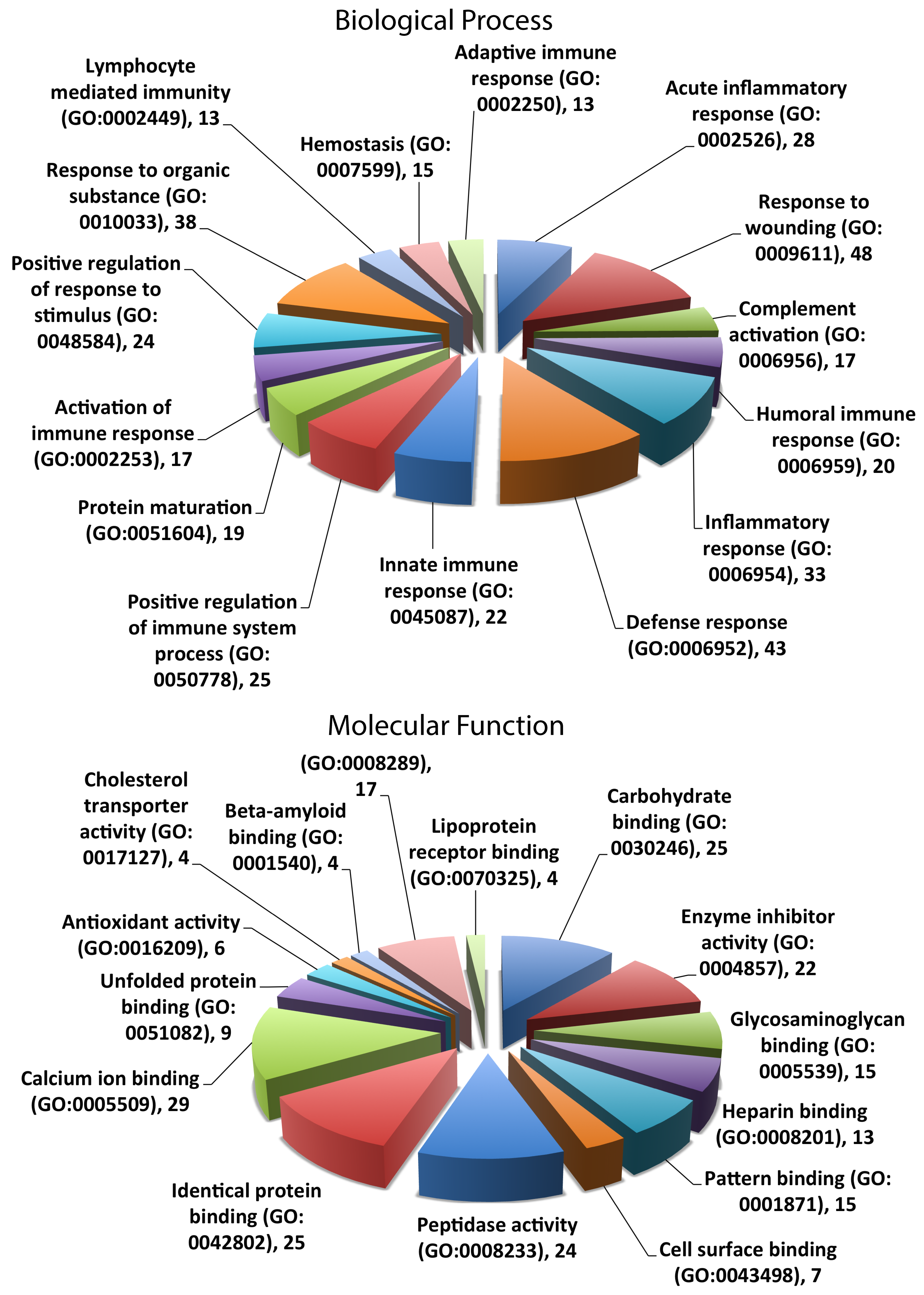

Supplement: S1 Fig — (TIF) [file pone.0139774.s001.tif]
